# Hemoglobin can Act as a (Pseudo)-Peroxidase *in Vivo*. What is the Evidence?

**DOI:** 10.3389/fmolb.2022.910795

**Published:** 2022-06-27

**Authors:** Abdu I. Alayash, Michael T. Wilson

**Affiliations:** ^1^ Laboratory of Biochemistry and Vascular Biology, Division of Blood Components and Devices (DBCD), Center for Biologics Evaluation and Research (CBER), Food and Drug Administration (FDA), Silver Spring, MD, United States; ^2^ School of Life Sciences, University of Essex, Wivenhoe Park, United Kingdom

**Keywords:** hemoglobin, ferryl hemoglobin, pseudoperoxidase, blood substitutes, fenton reagent

## Introduction

Reactive oxygen species (ROS) are known to provoke cellular and subcelluar changes which can ultimately disrupt normal physiology (Stadtman and Levine, 2000; Tsukahara and Yashiro, 2016). The generation of ROS in biological systems generally involves one electron reduction of oxygen (O_2_), mediated by a redox active metal center, forming superoxide (O_2_^•−^). Dismutation yields hydrogen peroxide (H_2_O_2_) that, on reduction, forms hydroxyl radicals (•OH) (Van Dyke and Saltman, 1996). The Fenton reaction involves iron (Fe^2+^) reacting with H_2_O_2_ to yield a hydroxyl radical (•OH) and a hydroxide ion (OH^−^) (Bystrom and Rivella, 2015) (Eq. 1).

$$Fe^{2+}+H_{2}O_{2}\to Fe^{3+}+•OH+OH^{-}$$ (1)

In biological systems, transition metals in some cases are integral part of macromolecules, such as DNA, proteins, and lipids. Hydroxyl radicals generated at these metal binding centers lead to site specific damage of these biomolecules (Van Dyke and Saltman, 1996).

Sadrzadeh et al. (1984) proposed that hemoglobin (Hb) can be considerd as a biologic Fenton reagent able to facilitate the production of hydroxyl radicals (OH•) from ROS and that these hydroxyl radicals are generated from the reaction of the iron of Hb with H_2_O_2_ (Lee and Lim, 2003). This was based on a simple *in vitro* experiment in which a O_2_^•−^ generating system (hypoxanthine and xanthine oxidase) produced hydroxyl-radical in the presence of Hb, in a dose-dependent fashion. This reaction was prevented by prior oxidation of Hb (methemoglobin) (metHb) or in the presence of CO or with addition of catalase (H_2_O_2_ scavenger) but not when superoxide dismutase (O_2_^•−^ scavenger) was present. Hb also increased the oxidation of arachidonic acid and membrane lipids of the red blood cells (RBCs). When haptoglobin (Hp) (plasma hemoglobin-binding protein) is added to the mix there was a considerable reduction in hydroxyl radicals and malondialdehyde formation. Based on these observations, Sadrzadeh et al., concluded that free Hb acts as a Fenton reagent, having the potential to catalyze hydroxyl-radical generation in areas of inflammation. This hypothesis however, was questioned and alternatively interpreted to be the reaction of H_2_O_2_ with iron released during heme degradation (Van Dyke and Saltman, 1996; Pupo and Halliwell, 1988; Gutterige, 1986).

The last three decades have witnessed increased commercial efforts in transforming cell-free Hb to an oxygen carrying (HBOC) therapeutic to be used in lieu of RBCs in transfusion medicine and this has naturally led to increasing opportunities for researchers to work on free Hb. This body of work has resulted in a more complete picture of the redox activity of Hb, as Hb gained the status of an “honorary enzyme,” i.e., a pseudoperoxidase (Reeder et al., 2004; Jia et al., 2007). Although, as we shall see, Hb has peroxidatic activity it is often termed a pseudoperoxidase as, in general, the first product of the reaction with H_2_O_2_, an oxo ferryl heme with a porphyrin radical cation, CpI, is ephemeral and does not participate in substrate transformation and because Hb damages itself on turnover with H_2_O_2_. Several other enzyme-like properties were also reported for Hb in recent years. For example, nitric oxide (NO) reacts avidly with Hb in a dioxygenase reaction (Gardner et al., 2006), and more recent controversial reactions such as S-nitrosylation of *β*Cys93 which results in the formation of the so-called SNO-Hb that can transport NO from lungs to tissues (Reynolds et al., 2007), nitrite reductase activity which results in NO production (Kim-Shapiro et al., 2011) were also reported.

### Pseudoperoxidase Activity of Hemoglobin

OxyHb spontaneously oxidizes under aerobic conditions to non-oxygen carrying ferric Hb (HbFe^3+^) (MetHb) in a process known as autoxidation (Eq. 2). The multistep pseudoperoxidative cycle of Hb is triggered by the reaction of Hb with H_2_O_2_. Central to this activity is the transformation of ferrous Hb (HbFe^2+^) to the highly reactive intermediates described in Eqs 3–6. In the presence of H_2_O_2_, a catalytic cycle is initiated to include the following steps: 1) Initial oxidation of HbFe^2^ (oxy/deoxy) to ferryl (HbFe^4+^) (Eq. 3), 2) autoreduction of HbFe^4+^to HbFe^3+^(Eqs 4, 5), and 3) reaction of HbFe^3+^ with an additional H_2_O_2_ molecule to regenerate the ferryl intermediate (Eq. 5), now accompanied by a protein-based radical (Eq. 6) (Jia et al., 2007; Kassa et al., 2015). Ferryl Hb, depending on pH, can be a highly reactive species, due to its high redox potential (E1/2° ∼1.0 V), and can oxidize other biological molecules. It has been proposed that at approximately pH 4 ferryl Hb can be protonated and generate a species that has characteristics of the highly reactive hydroxyl radical (Eq. 4). Ferryl Hb can be measured by conventional spectrophotometric methods while the protein-based radical associated with the ferryl species can be measured by electron paramagentic resonse (EPR) spectrometry (Vlasova, 2018).

$$HbFe^{2+}O_{2}\to HbFe^{3+}+O_{2}^{•-}$$ (2)

$$HbFe^{2+}+H_{2}O_{2}\to HbFe^{4+}=O^{2–}+H_{2}O$$ (3)

$$HbFe^{4+}=O^{2–}+H^{+}\to HbFe^{4+}=OH^{–}/HbFe^{3+}-OH^{•}$$ (4)

$$HbFe^{4+}=OH^{–}/HbFe^{3+}-OH^{•}+H^{+}+e^{–}\to HbFe^{3+}+H_{2}O$$ (5)

$$HbFe^{3+}+H_{2}O_{2}\to P^{•+}HbFe^{4+}=O^{2–}+H_{2}O$$ (6)

(The protein radical cation, P^•+^, deprotonates to yield the stable EPR detectable P^•^)

One of the hallmarks of Hb-mediated oxidative changes is the irreversible oxidation of a number of amino acids in the “hotspot” region and the formation of a heme cross-link to the protein that have been attributed to the ferryl/ferryl radical. In the presence of H_2_O_2_, oxoferryl heme (HbFe^4+^ = O^2−^), together with its protein radical (P^•+^HbFe^4+^ = O^2−^) are formed (Eqs 3–6). Once formed this radical irreversibly oxidizes *β*Cys93 to cysteic acid and interacts with other “hotspot” amino acids that are highly susceptible to oxidative modifications (Jia et al., 2007). In addition, a covalent heme-to-protein bond has been detected by HPLC in the reaction of Hb and H_2_O_2_. This adduct has been recognized as a valuable biomarker for the peroxidatic activity of Mb and Hb (Wilson and Reeder, 2022). Hb and myoglobin (Mb) have therefore been decribed as pseuopeoxidases as they, unlike true preoxidases, undergo self-inflicted changes at the protein and protein/heme levels (Vlasova, 2018). Hp, one of the most important naturally occuring antioxidants in human plasma, has been shown recently to suppress Hb peroxidative activity by stabilizing HbFe^4+^, suppressing the radical-generating steps after exposure to oxidants such as H_2_O_2_ (Alayash, 2011; Mollan et al., 2014). Ferryl Hb stabilization is accompanied by a significant increase in the concentration of ferryl protein radical on some tyrosine residues (Cooper, 2013; Svistunenko and Manole, 2019).

### Pseudoperoxidase Activity of Hemoglobin *in vivo*

An early experiment using electron paramagnetic resonance (EPR) designed to detect endogenous defences against ferryl/ferryl radicals was reported for a rabbit model; rabbits unlike humans maintain an effective ascorbate reducing systems in blood. In these experiments, animal blood was exchange transfused (20%) with cell-free Hb stabilized in tetrameric form developed as a blood substitute. It was found that metHb levels in circulation were reduced to oxyHb by a slow process (t_1/2_ = 1 h), with no globin-bound free radicals found in the plasma of these animals. This is because redox defenses were fully active as part of a multifunctional plasma ascorbate system able to remove key precursors of oxidative damage (Dunne et al., 2006). Accordingly, these experiments demonstrated that ascorbate was able to effectively reduce plasma metHb, ferrylHb and its associated globin radicals. The ascorbyl free radicals detected by EPR were efficiently re-reduced by the erythrocyte membrane-bound reductase (which itself uses intraerythrocyte ascorbate as an electron donor) (Dunne et al., 2006; Sibmooh et al., 2008).

In a separate EPR study the same group (Svistunenko et al., 1997) detected a protein based radical at ∼1 μM concentration in venous blood samples from healthy human adults. This radical was identical in line-shape and power saturation charactristics to that generated *in vitro* experiments through the reaction of ferric Hb with H_2_O_2_. Chromatographic and mass spectroscopic analyses of these samples confirmed the presence of oxidatively modified protein-heme products which is known to incrase significantly during acute oxidative stress (Vollaard et al., 2005). It is interesting to note that, even within the protective environment of RBCs the characteristic EPR protein radical signal, representing oxidative damage to the heme and by implication the oxoferryl species was detected. Similar ferryl driven protein-heme modifications were also reported in acute kidney dysfunction following rhabdomyolysis (Wilson and Reeder, 2022) and in cerebral spinal fluid following subarrachnoid hemorrhage (Reeder et al., 2002).

Spectrophotometric, and LC-MS/MS investigations of atherosclerotic lesions of the human carotid artery and hemorrhagic cerebrospinal fluid from preterm infants led recently to the discovery of oxidized Hb intermediates and cross-linked globin chains in these samples. The vascular pathophysiologic impact of oxidized Hb and the resultant peptides were assessed by measuring endothelial integrity, the activation of endothelial cells and the induction of proinflammatory genes. The presence of peptide fragments and ferryl Hb (as measured by photometric and Western blot methods) confirmed that Hb oxidation products triggered endothelial cell dysfunction (Posta et al., 2020). In a follow up study the same group used specific monoclonal anti-ferrylHb antibody and found that ferrylHb was localized extracellularly and also internalized by macrophages in the human hemorrhagic complicated lesions, this followed by upregulation of heme oxygenase-1 and H-ferritin and accumulation of iron within lysosomes as a result of heme/iron uptake (Potor et al., 2021).

One of the most revealing and biologically relevant experiments on the psuedoperoxidase activity of Hb came from observations made in sickle cell disease (SCD) in humans and in animal models. Sickle cell Hb (HbS) oxidizes faster and its ferryl persists longer in solutions than ferryl HbA (Kassa et al., 2015). A recent study shed some light on the question of how HbS provides protection against malarial parasitic infection in SCD. The study found that ferryl Hb inhibited actin polymerization in RBCs -infected malaria, thereby preventing the malarial parasites from creating their own actin cytoskeleton within the host cell cytoplasm (Cyrklaff et al., 2011; Alayash 2018).

The oxidation of intracellular Hb and its contribution to the RBC-derived microparticles (MPs) formation was recently investigated in the homozygous Townes-sickle cell (Townes-SS) mice model. Photometric and proteomic anlayses of the MP content showed the presence of considerable levels of Hb oxidation intermediates (ferric/ferryl). This experiment also revealed the degree of *β*-globin posttranslational modifications (PTMs), that included irreversible oxidation of βCys93 and the ubiquitination of *β*Lys96 and *β*Lys145 (Jana et al., 2018). Critically, ferryl Hb but not hemichromes was found to induce the complex formation with band 3 and RBC membrane proteins consistent with early *in vitro* reports (Welbourn et al., 2017). When MPs obtained from Townes-SS mice that were fed on a diet rich in hydroxyurea (HU), fewer PTM modifications were found on Hb. *In vitro*, hydroxyurea (NO producing molecule) reduced the levels of ferryl Hb and shielded its target residue, *β*Cys93, by a process of S-nitrosylation (Jana et al., 2018).

A follow up clinical investigation using MPs from the blood of SCD patients who were either on or off HU treatment, and from ethnic matched controls was carried out recently (Strader et al., 2020). Blood samples from subgroup of patients were studied prior to initiation and during HU treatment were also analyzed by mass spectrometry-based proteomics. These analyses revealed that band 3 and its interaction network involved in MP formation samples from SS patients exhibited extensive protein phosphorylation and ubiquitination in SCD patients than in controls. Samples from patients who were treated with HU showed little or no oxidative PTMs similar to those samples from the control group. (Strader et al., 2020).

## Summary and Conclusion

Ferryl Hb has been detected in several *ex-vivo* and *in vivo* model systems; in atherosclerotic lesions of carotid arteries, in blood from mice and SCD patients, and in blood from SCD patients infected with malaria. Unique cellular and in some instances subcellular injuries have been attributed to the ferryl Hb’s redox reactivities. Collectively, these studies (decribed above) established a fundamental role for cell-free Hb and Mb in oxidative cellular injury. These events are mediated through the redox transition of Hb to higher oxidation states, leading to oxidative modification/damage to biological molecules.
